# Book Reviews

**Published:** 1809-07

**Authors:** 


### CRITICAL ANALYSIS
### OF THE
### RECENT PUBLICATIONS
### ON THE
### DIFFERENT BRANCHES OF PHYSIC, SURGERY, AND MEDICAL PHILOSOPHY.

---

*Medico-Chirurgical Transactions, published by the Medical and Chirurgical Society of London. Vol. I.*

To give a just view of the nature of the Society to which we are indebted for this Volume, we shall transcribe part of the Preface.

"On submitting to the public the first volume of the Transactions of the Medical and Chirurgical Society, the President and Council, to whom the management of its affairs is entrusted, feel it to be their duty to state the nature and objects of this Institution, and the claims which it possesses to professional patronage.

"The want of a Society, founded upon liberal and independent principles, and conducted with the propriety and dignity which are worthy of the medical profession, had been long acknowledged; and a few physicians and surgeons in the year 1805, held a meeting for the purpose of considering the best means of obviating it. They invited many gentlemen of eminence to join them, and thus a Society was formed, which they soon had the satisfaction to see, comprised a very respectable portion of the professional rank and talent of the metropolis.

"The present volume is composed of papers which have been communicated to this Society, and read at its meetings. The president and council submit it to the consideration of the medical public, not without the hope, that it will support the claim of respectability and usefulness, which they are desirous that it should possess.

"The papers which come before the Society have necessarily various degrees of value; and in considering their merits with a view to publication, it is wished equally to avoid the extremes of fastidiousness, and want of discrimination. Brilliant discoveries in medicine and surgery, or the branches connected with them, are seldom made; but the observing practitioner has various opportunities of improving the profession, by attention to the facts which come daily within his view, and by the management of the materials which are already in his possession.

"The varied forms of disease, whether medical or surgical, and the modes of treatment which may be found adequate to their removal, are subjects concerning which the Society necessarily feels the highest interest. Cases having a fatal issue, however, are often not less instructive than such as terminate favourably. They frequently tend to point out more accurately the plan to be pursued in the treatment of similar complaints; they afford valuable information relative to the probable causes of failure; and when dissection is permitted, they throw light on the more intimate nature and modification of the disease.

"The operative part of surgery opens a field of considerable interest and extent, and the number of gentlemen connected with hospitals in London, who are members of the Society, gives it the prospect of being able to communicate to the public, some valuable observations and improvements in this important branch of the profession."

"The want of a society formed on liberal or independent principles," is not so apparent to us as to the writers of the above. The London Medical Society has always appeared to us founded on such principles. That parties have occasionally been formed in that as well as in in all other associations, can hardly be questioned; but we know of none which have disturbed its general harmony, and precluded the admission of either members or papers for publication. It will, perhaps, be said, that both have been too indiscriminately admitted. In answer to this, we must remark, that till the encouragement given within the present century to a popular Journal, there was no regular record for many facts, which, if not brilliant in their issue and recital, were at least of sufficient importance to be recorded. It may be urged, that the last volume of Memoirs which has been published since the period alluded to, does not show more circumspection in its choice of papers. This must be admitted, and, probably, the erection of a rival institution may be serviceable to both.

The plan of the two Societies seems similar; each publish volumes when they have collected a sufficient number of interesting papers. Each read a paper when one is offered. Each admits conversation without allowing it to degenerate into debates. Each proposes the augmentation of its library; and each solicits communications from whatever quarter. In the new Society, the Preface remarks, "The union of gentlemen of both branches of the profession, affords a greater facility of obtaining accurate information on many points of practice, than could have been derived from a society composed of either physicians or surgeons only." By the latter part of this sentence, we were fearful apothecaries, whose opportunities of information are at least equal to either of the other two branches, were excluded; but in perusing the list which follows, we are glad to find this is not the case. We do not, however, perceive any names on the council, but those of physicians or surgeons.—Having given this general account of the Society, we shall proceed to offer an abstract of their "Transactions."

ARTICLE 1.—*A Case of Aneurism of the Carotid Artery.* By ASTLEY COOPER, Esq. F. R. S. Surgeon of Guy's Hospital.

To do justice to the author, we shall transcribe the account of the operation, which will give a sufficient insight into the disease.

"The tumour at this time reached from near the chin beyond the angle of the jaw, and extended downward to within 2 ½ inches of the clavicle. I made an incision two inches long, on the inner edge of the sterno-mastoid muscle, from the lower part of the tumour to the clavicle, which laid bare the omo and sterno-hyoideus muscles, which being drawn aside toward the trachea, exposed the jugular vein. The motion of this vein produced the only difficulty in the operation, as under the different states of breathing it sometimes presented itself to the knife, tense, and distended, and then as suddenly collapsed. Passing my finger into the wound to confine that vein, I made an incision upon the carotid artery, and having laid it bare, I separated it from the par vagum, and introduced a curved aneurismal needle under it, taking care to exclude the recurrent nerve on the one hand, and the par vagum on the other. The two threads were then tied about half an inch asunder, being the greatest distance to which they could be separated; I thought it proper not to run the risk of a hemorrhage by dividing the artery, as I was fearful the ligatures would be thrown off by the force of the heart, and the distance was too small to allow of any means being used to prevent it. As soon as the threads were tied, all pulsation in the tumour ceased, and the operation being concluded, and the wound superficially dressed, she rose from the chair in which she sat during the operation, and was immediately seized with a fit of coughing, which I thought would have terminated her existence. This seemed to arise from an accumulation of mucus in the trachea, which she could not expel; it continued about half an hour, when she became more tranquil.

"*Saturday, Nov.* 2.—Mr. Owen, who had sat up with her, reported that she had slept six hours during the night, but was now and then disturbed by her cough. The pulsation in the tumour has not returned; that in the brain has ceased, and there is no appearance of diminution of nervous energy in any part of the body."

On the same night, leeches were applied to the head to relieve her of pain in that part; next day her cough better, pain gone; the following night slept 6 hours.

The following day mending, but cough returned; increased on Tuesday; the succeeding day, Wednesday, so violent as to force a few drops of venous blood from the orifice.

Thursday, the symptoms mended.

Friday, general irritation, and the left leg and arm somewhat paralytic. Head free from pain.

Saturday, all the symptoms improved. Wishes to eat, but unable to swallow solids.

Monday, symptoms mended.

Tuesday, the paralytic arm recovered.

The ligatures, with the intervening piece of artery, came away with the dressings; all the symptoms mended till Sunday the 17th of November, the seventeenth day from the operation. From this day, the symptoms of irritation increased, with an ill condition of the wound; on the 21st she died.

By examining the part subjected to the operation, it appears that "the cause of her death was the inflammation of the aneurismal sac and the parts adjacent, by which the size of the tumour became increased so as to press on the pharynx and prevent deglutition, and upon the larynx, so as to excite violent fits of coughing, and ultimately to impede respiration.

"A similar event, however, may be in future prevented, by performing the operation when the tumour is small, and pressure has not been made by it upon important parts, or if it is of considerable size, as in this case, by opening the tumour and discharging the coagulum, as soon as inflammation appears."*

Permission

"* Since this paper was read to the Society, another case has occurred which has terminated successfully, and will be given at the end of this volume."

Permission not being obtained to open the head, the cause of the paralysis could not be discovered.

If we may be allowed to make any remarks on this case, of which we may truly say, "*quem si non tenuit, magnis tamen excidit ausis,*" we should be induced to regret that so important an operation was undertaken under circumstances so unfavourable. Considering the rank of life of the patient, it was hazardous to trust her under such circumstances any where but in the hospital. The very short time between the first interview and the performing of the operation, might, perhaps, have been usefully employed in preparing the constitution for it, or the first symptom of inflammation might, if not anticipated, have at least been met by copious bleeding. We are not urging that these or any other means could have saved the patient, or what is still more to the purpose, that she could have lived without submitting to the operation: but we feel it impossible to restrain our expression of regret, that so good a thing did not succeed perfectly. We shall, however, do all we can to reconcile ourselves and our readers to the event, by noticing in this place, though out of order, a

*Second Case of Carotid Aneurism.* By Astley Cooper, Esq. F. R. S. Surgeon to Guy's Hospital.

This case is introduced by a concise but ample apology for performing such an operation. The subject was a porter on the Quays, 50 years of age, the disease of about 8 months standing. The tumour when first observed, 6 months before the operation, was about the size of a walnut, just under the angle of the left "jaw, and extending from thence downwards to the thyroid cartilage. It was accompanied with great pain on the left side of the head, which began about five months ago, and was attended with a sense of pulsatory motion in the brain. The tumour affected his speech, so as to make him extremely hoarse; and he had more recently a cough, attended with slight difficulty of breathing, and which seemed to be the effect of the pressure of the swelling on the larynx. His appetite was sometimes affected by it; for three or four days he eat heartily, and then for many lost his relish for food. He had a sense of coldness succeeded by heat in his left ear, and he often became sick when eating, but did not vomit. Upon attempting to stoop at any time from that period, he had an insupportable feeling as if his head would burst; a giddiness; loss of sight; and almost total insensibility.

"The left eye, which had for some time been gradually closing, appeared now not above half as large as the right; yet its power of vision was equally perfect.

"A blister was at this time ordered to be applied on the head by Dr. Hamilton, which lessened his pain. A month ago he applied another with the same relief; but it lasted only for a few days. He continued at work until the day previous to the operation.

"The dilatation of the carotid artery was seated just below the angle of the jaw, and about the acute angle which is made by the great great division of the common carotid. The tumour was about the size of a pullet's egg, and prominent in its middle.

"The pulsation of the aneurism on the day of the operation was remarkably strong; when the sac was emptied by pressure on the artery below, the tumour sprang to its original size with one contraction of the heart."

In this operation, the artery was divided between the ligatures. No unfavourable circumstances occurred to interrupt the healing of the parts or the health of the patient, who, within 3 months after the operation, was enabled to pursue his occupation without injury or danger.

"The result of this case (says Mr. Cooper) afforded me a degree of pleasure which compensated for the disappointment I felt in the issue of the former. In a professional point of view, it was highly desirable to ascertain the possibility of saving life in a case which had hitherto proved generally fatal; and I could not but feel more than common interest in the fate of a man, who, although he well knew that the trial was new, and the risk considerable, never betrayed the smallest signs of apprehension.

"Near eight months have now elapsed since the operation was performed, and he has returned to his former employment without any diminution of his mental or corporeal powers, excepting the lessened action of the temporal and facial arteries on the side in which he was operated. The tumour has disappeared, and he has not been since subject to that pain in the head, by which he had been so much distressed prior to the operation.

"This aneurism, from the depth of its situation, was, I believe, seated in the internal carotid artery, and this led me to hope that the regurgitation of the blood, although at first sufficient to produce a slight pulsation in the tumour, would not continue to support its growth, because as the internal carotid passes through a foramen in the skull, a little above the swelling, it could not dilate at that part to bring down any additional quantity of blood into the sac; so that its first effect was likely to be as great as any it could produce. But if the aneurism had been of the external carotid artery, owing to the number of communicating vessels, I should not have been equally sanguine in my expectation that the pulsation would have ceased, as I have known two instances, one of a wounded radial artery, and the other of aneurism of the anterior tibial, in which the tumour continued to grow by anastomosis, after the arteries had been tied above the swellings."

ARTICLE 2.—*Case of Violent and Obstinate Cough, cured by a Preparation of Iron.* By CHRISTOPHER STANGER, M. D. Gresham Professor of Physic, and Physician to the Foundling Hospital.

THIS is a very interesting paper, on account of the accurate description of the case, and the pathological remarks of the author. The remedy, however, was very similar to Griffiths's celebrated mixture, mixture, which that practitioner conceived was a cure for phthisis pulmonalis.

Article 3.—*Some Observations relative to the Treatment of the Hooping Cough.* By Richard Pearson, M. D. F. A. S.

It is some consolation to find that the rising generation will be freed from those miseries which we have undergone, and some of us inflicted on others. Emetics, since the days of Dr. Fothergill, have till lately been considered the remedy for hooping-cough. The author of the present paper proposes, after the accumulation of phlegm has been brought away by an antimonial vomit, a medicine prepared of opium, ipecacuhana, and prepared natron.

We have no doubt of the efficacy of this remedy; but this does not prevent our making a few remarks. In the early stage of the disease, cathartics will be found very useful, and *nostro periculo* may entirely supersede the use of emetics. In the progress, the symptoms must be attended to as in other complaints, and the remedies applied according to each; but the history of every case and of every remedy, will be very imperfect if the season of the year, temperature of the weather, and situation of the person in all other respects, are not most carefully registered throughout the whole. We ought to add, that our author has not overlooked the necessity of attending to all the symptoms, or of varying the practice according to them.

*On a Diminution (in consequence of disease) of the Area of the Aperture, by which the left Auricle of the Heart communicates with the Ventricle of the same Side.* By John Abernethy, Esq. F. R. S. Assistant Surgeon to St. Bartholomew's Hospital, and Vice President of this Society.

Malformations of the heart, are of all others the most vexatious, because we witness the sufferings of the patient, are constantly anxious that we may be mistaken in our prognosis, and certain that if right, we can offer no relief. The most remarkable circumstance in this paper, is the accuracy with which Mr. Abernethy conjectured, during life, the exact seat of the disease, which, in an organ whose cavities and their communications are so numerous, and whose actions so much influence the whole vascular system, is oftentimes extremely difficult.

" Notwithstanding, says he, the respiration was laborious and frequent, it still appeared too free to admit of the supposition, that the left cavities of the heart received the blood in such small portions, in consequence of an impediment to its transmission through the lungs. I therefore conjectured, that a mechanical impediment prevented the left ventricle from receiving its due quantity from the auricle, and I could suggest no other kind of impediment, but some polypous growth or unnatural tumour. The symptoms increased in severity for the course of a month, when the patient died."

On examination, a passage from the left auricle to the left ventricle was found of an eliptical form, situated between the sides

 of of two pointed productions of the mitral valves, and not greater than would admit the passage of a moderate sized bougie, adapted to the form of the aperture. The following are the author's reflexions on this case.

"The annulus venosus is a strong callous ring by which the auricles communicate with the ventricles. If the left cavities of the heart were injected to the utmost, the ring would be probably about one inch and a half in diameter. From this circle arises that duplicature of the membrane of the heart, called the valvula mitralis, into the pointed edges of which the chordæ tendineæ are inserted. Now, if the fleshy columns which proceed from the middle of the heart are irritable, and frequently draw these tendinous chords, they will cause the two sides of the valve to approximate in a linear direction. Nay, as the base of the valve arises from the annulus venosus, they will even produce an approximation in the sides of that aperture, and thus gradually tend to effect the kind of contraction which I have described. One cannot, however, suppose the closure of the opening to arise from such force alone, but from an alteration of structure and a new formation of parts according with the effect which this force would occasion. It might be suggested, that this was the mere effect of inflammation; but inflammation might cause thickening, or perhaps ossification of the valve, as we frequently see, without producing this contraction. When, however, inflammation takes place in the mitral valve, it will in some degree be extended to the tendinous chords which terminate in its points, and by these means to their fleshy columns, rendering them irritable, and thus producing the effect of approximating the sides of the valve in different degrees.

"In the dissection which has been related we found evidences of inflammation, but not considerable in degree. That the action of the heart tends to diminish the aperture between the auricle and ventricle, is, I think, evident from examining the heart of an animal which has been bled to death, when it is found in its utmost state of contraction; and then it is difficult to pass even the little finger from the auricle into the ventricle. I have, as I before said, met with various degrees of contraction in the aperture by which the two cavities of the left side of the heart communicate, in the ordinary course of dissections, and in some instances even to the extent described in the foregoing case. I knew nothing, however, of the symptoms which took place during life.

"A late instance, however, has occurred in St. Bartholomew's Hospital, in which the symptoms were noted during life, and the morbid appearances after death. The dissection was made by Mr. Barnes, the House Surgeon, whose diligence and talents are, I think, likely to render service to the profession, and whose account of the dissection I here subjoin.

"It will, however, be previously proper to give a brief account of the symptoms which the disease occasioned.

"The

"The patient was a woman of a thin and small form, and about thirty-eight years of age. I should unnecessarily occupy the attention of the society, was I to detail all the particulars of the case, as they were recorded, during the patient's life. Many circumstances related to the state of the stomach and bowels, and to that of the nervous system. It seems only necessary to mention those which appear more immediately to have arisen from the mechanical impediment to the circulation of the blood; and there were (as in the former case) an extremely small and frequent pulse, a purple tint of the skin, particularly in the face; but there was no œdema, as in the preceding case. The smallness and frequency of the pulse increased, as the disease advanced, so as to render it difficult to feel and count it."

In the former case, we should remark, that the right cavities were found distended with blood. In this, the right cavities were empty. The left auricle was filled with blood, coagulated in layers; and its muscular parietes thickened. The annulus venosus appeared like a slit, an inch in length, and one eighth in breadth, with an irregular hardened edge, cartilaginous, white, and with difficulty admitting the fore finger. On cutting off the apex of the ventricle, the parts below had nearly a similar appearance, the mitral valve being much thickened, opake, and of the hardness of cartilage.

"In this case, says Mr. Abernethy, there was a much greater disease of the valve, than in the one which I first related. It is useful to shew the different circumstances in which the contraction takes place. I repeat that it is, as far as my observation enables me to determine, a frequent occurrence in various degrees. We also meet sometimes with considerable disease of the valves with but little contraction in the passage between the auricle and ventricle; and we meet with great contraction sometimes, where but little inflammation seems to have existed. These observations shew, that the contraction does not depend on inflammation alone, and led me to suppose, that it was produced in the manner that I have stated in a former part of the paper."

A very curious case of diseased ovary follows, which occurred in Mr. Hurlock's practice.

Article 5.—*An account of a very peculiar Disease in the Heart.* By David Dundas, Esq. Serjeant Surgeon to his Majesty.

This truly valuable paper contains an account of several cases, in which, under rheumatic fever, the disease has been suddenly transferred to the heart. The symptoms in that organ are peculiarly acute, and the palpitation often so violent, as to be distinctly heard, and even to agitate the bed in which the patient lies, with such violence, that the pulse may be counted by the motion of the curtains. The respiration is painful. At the same time, the pulse is small, quick, and sometimes irregular; in some instances, weak, but more commonly hard. As the disease advances, the legs swell, sometimes the abdomen, and the patient dies after wretchedly ly lingering for an uncertain period. All the cases seen by the author were young subjects, six males, and three females. In all that have been opened, the heart was enlarged in different degrees, principally the left ventricle, but the increase was not in the muscular fibres, which were found unusually pale, even soft and tender in their texture. The pericardium adhered to the heart in every instance but one, in which water was found in it.

A case with many similar symptoms is related by Mr. Thomas, in which an irregular excrescence of the nature of a polypus was found attached to, and nearly occupying the whole of one of the valvulæ mitrales. The left side of the heart was somewhat larger than natural. Another by Dr. Baillie, which occurred in the year 1770. The pericardium adhered to the le t ventricle, which was thinner than the right. Where the pericardium did not adhere, a small quantity of water was found within it. Another by the author of the paper, in which the heart was found of an unusual size, its substance pale and tender, the pericardium every where adherent. The valvulæ mitrales edged with a substance of a spongy appearance; perhaps, says the author, coagulable lymph.

A case follows, related by Dr. Pemberton. The patient was a male, 36 years of age, and subject to acute rheumatism, of which he had a severe fit immediately preceding the disease in the heart. The symptoms of an affection about the heart were very violent, but somewhat relieved by cordials, after which a small quantity of blood was taken, which was not buffy. The symptoms gradually subsided under the use of digitalis and cicuta, a low diet, and a seton in the region of the heart. Two cases have occurred to Dr. Marcet of rheumatism transferred to the heart, with similar symptoms. They proved fatal, and in each the heart was found enlarged. The last case related is by Mr. Dundas. The patient was a female, 29 years old. Besides the symptoms about the heart, ascites followed. The heart was found to occupy the greater part of the left side of the thorax, the lungs strongly adherent to the pleura.

Of all the cases related by Mr. Dundas, it is remarkable, that only two were above 22 years old. About the year 1781, we recollect a similar case at St. Bartholomew's Hospital. The subject was a boy about 18, and the third of his father's children who had died under similar symptoms, at the same age. We shall forbear to offer any opinion on the cause.

Article 6.—*On the Gelatine of the Blood.* By John Bostock, M. D. Liverpool.

The labours of Dr. Bostock in that most valuable inquiry, animal chemistry, has appeared on many occasions. The present is not less important, though the result may not confirm any generally received opinion. According to Parmentier, Birkbeck, Fourcroy, his translators Thompson and Delametherie, blood is supposed to consist of fibrine, albumen, gelatine, red globules, soda, soda, some neutral and earthy salts, a small portion of sulphur, and a peculiar phosphate of iron, all held in solution by a large quantity of water. The crassamentum is composed principally of fibrine and red particles. To the red globules the iron is attached, which is supposed to impart the red colour to the blood. The albumen, jelly and salts, dissolved in water, have been considered as constituting the serum. The albumen is procured by subjecting the serum to heat. If after its coagulation by that process it is cut into small pieces, and digested in water, the other ingredients will be suspended in the water, and leave the albumen tolerably pure.

"By evaporating the water we obtain the jelly, but it is unavoidably mixed with the salts, from which it does not appear possible entirely to separate it; this object can only be in part accomplished by a slow evaporation of the water, in consequence of which, a portion of the salts will assume their regular chrystalline form, and may be thus removed from the mass. The small quantity of sulphur which exists in the blood, appears to be united to the albumen; it has, however, never been obtained in a separate form, and its existence must be regarded as somewhat problematical. That part of the serum, which remains fluid, after the albumen has been coagulated by heat, to which the name of *jelly* or *gelatine* has been applied, is the last of the constituents of the blood, the presence of which has been distinctly ascertained, and it is the one to which in the present paper I propose principally to direct my attention."

The author next proceeds to give an account of the opinions of such authors as have expressly or incidentally written on the subject, before he offers the result of his own experiments. We shall pass over all that has been said before the late improvements in chemistry, or at least, till the researches of modern physiologists produced a more accurate distinction in the language of each.

The first person who uses the term, "serosity," as a part of the blood, is Senac, in his *Traité du Cœur*; but, as Dr. Bostock sufficiently proves, Senac had no meaning in the word, different from the *serum*, never considering it as a part distinct from the rest. De Haen seems to have been the first person who used the term gelatum as a part of the blood, but there is no satisfactory proof that he meant jelly, strictly so called, by such a term, for the manner in which he procured it was incompatible with such an effect, even if jelly had really existed in the serum.

Haller, in his physiology, speaks of the serum as consisting of water, mucus, and jelly: but there is reason to believe he used the latter expression very indefinitely; of this one expression quoted by Dr. Bostock, *seri pars quæ cogitur vel gelatina*, is sufficient proof.

Dr. Butt, in an inaugural dissertation, published at Edinburgh in 1760, divides the serum into a coagulum and watery part, but evidently evidently shows that he considers the coagulable lymph and albumen as the same substance.

Cullen appears to have entertained the same opinion. He made use of Senac's term, serosity, our author remarks, to distinguish that part of the serum which was separable from the albumen.

Hewson was more correct in distinguishing the coagulable lymph from the serum. He used the term serosity in the same sense as Cullen, fancying that he is following Senac, in which we have shown that he was mistaken. He considered that this serosity or watery part of the serum contained the salts diffused in the blood, and remarks, that by evaporation, a mucilage which it contains, is hardened so as to resemble the mucus spit up by the blood, and dried. Such was the state of the question when the chemists engaged in the enquiry. "If serum, says M. Fourcroy and Vauquelin, after being mixed with half its weight of water, be exposed to heat, it is in part coagulated; and the portion of liquid which is not coagulated contains gelatine, which gelatinizes by cooling." In a future paper, they seem to explain themselves with more caution, saying only, that a slightly turbid fluid may be separated from coagulated albumen, which by evaporation and cooling, concretes into a substance having all the characters of jelly.

In 1794, a still more particular account is given of this substance, by Parmentier and Deyeux. But though they seem disposed to admit the result of their countrymen, yet it appears, that with all their diligence, they procured only a substance glutinous to the touch, and which when dried composes a hard transparent film. But this is not the true character of jelly, which should be fluid under heat, and concrete with cold. Dr. Bostock also remarks, that they offer no chemical tests to prove the existence of gelatine.

Mr. Hunter, about the same time, was engaged in experiments on the blood. With little or no knowledge of chemistry, we can only expect that he should faithfully narrate what he saw, and draw no conclusions without explaining the sources from which he derives them. After describing the serum as consisting of albumen, and a fluid not coagulable by heat, he adds, that he found the latter might be precipitated by Goulard's extract, and the precipitate he conceived was the same as albumen. In this he was evidently mistaken, Dr. Bostock having lately discovered that the acetate of lead is the proper test for mucus.

Since this period, the chemical writers have followed each other, in representing jelly as one of the constituent parts of the blood. Mr. J. Bell, in his anatomy, makes some objections to the general doctrines of other writers, but he confounds coagulation with gelatinization, and his mode of writing on this subject is so confused throughout, that it is not easy to develope his meaning.

Having thus noticed the opinions of others, Dr. Bostock reminds minds us of those tests with which he formerly favoured the public for examining the constituent parts of animal matter. Our readers will recollect his account of the three substances most nearly resembling each other in their visible properties, and which being found in most parts of the body, he calls primary, viz. albumen, jelly, and mucus.

" The distinguishing characters of the first are, its being coagulable by heat, and by the oxymuriate of mercury. The second is liquefied by heat, and becomes solid again by cold ; it is not affected by the oxymuriate of mercury, but is precipitated from its solution by tan ; while mucus is neither coagulated by heat, nor has the power of concreting by cold ; it is not affected by the oxymuriate of mercury, nor by tan, but it is copiously precipitated by the acetate of lead.

" In order to ascertain the nature of the uncoagulable part of the serum, I exposed a quantity of it for some time to the heat of boiling water; it concreted in the usual manner into a solid mass, but upon being divided into small pieces, and laid upon an inclined pane of glass, a brownish liquor oozed from it. The pieces of serum were afterwards digested in boiling water, which became tinged of a brown colour, owing to some substance previously contained in the serum which it had carried off. The fluid which oozed from the coagulated serum, and the water in which it had been digested, were added together. To a portion of it a small quantity of the solution of the oxymuriate of mercury being added, it became milky, and a precipitate was formed ; it was also rendered opake by being for some time exposed to the boiling temperature. Hence I found that it still contained some uncoagulated albumen ; and in order more effectually to separate it, I diluted a quantity of serum with six times its bulk of water; to this I added the solution of the oxymuriate of mercury, until no farther precipitation could be perceived, and placed the compound in the water bath. The coagulum was by this process rendered considerably firmer than when heat only had been employed, and the liquor remained nearly transparent; it was passed through a filtre, and now no precipitate could be obtained by the addition of the infusion of tan. A quantity of the water, in which coagulated serum had been digested, was slowly evaporated; when the greatest part of the water was separated, it was suffered to cool, but no appearance of gelatinization was perceptible. The evaporation was then continued to dryness; a tenacious film of animal matter was left behind, which did not in any respect resemble dried jelly, and which was with difficulty re-dissolved by the addition of more water. These experiments were several times repeated, and the results were essentially the same, so far at least as affected the conclusion to be drawn from them. It is necessary however to remark, that in trying different specimens of serum, there was a considerable difference perceptible in the readiness with which the albumen was separated from the uncoagulable part; in some instances stances a single operation was sufficient, while in others it was necessary to repeat the addition of the oxymuriate of mercury and the boiling four or five times, until the liquor which passed through the filtre was entirely freed from the uncoagulated mass.

" From these experiments I felt myself justified in concluding: First, That when diluted serum is completely deprived of albumen, which is proved by its no longer yielding a precipitate, upon being boiled with the oxymuriate of mercury, it is not affected by the infusion of tan, Secondly, That the animal matter contained in serum, which is not coagulated by the operation of heat or the oxymuriate of mercury, does not possess the property of concreting by cold. Whence we may infer, in the third place, That that part of the serum which is not coagulable by heat, does not possess the properties which are essential to jelly, either physical or chemical."

It is hence shown that the precipitable part of the serum left after the separation of the albumen can only be mucus, mixed perhaps with some small portion of the salts. Dr. B. remarks, that he has since had an opportunity of examining the fluid in a diseased spine, in hydrocephalus internus, and in hydrocele, in neither of which could he detect any jelly.

The paper concludes with a short apology for differing from so many respectable authorities. It is shown however that the operative chemists do not say that the substance they obtained *concreted by cold*, which is the true property of jelly strictly so called. Mr. Hunter's experiments, as far as they go, exactly confirm Dr. Bostock's conclusion, the precipitate by acetate of lead being the true test of mucus. Dr. Brikbeck and Mr. Allen, indeed, speak of tan as throwing down a copious precipitate from the serosity; but as this fluid was obtained by pressing it from serum that had been exposed to heat, the author infers that it still contained a portion of uncoagulated albumen, which would be acted upon by the tan.

ARTICLE 7.—*Account of the Effects produced by a large Quantity of Laudanum taken internally, and the means to counteract those Effects.* By ALEXANDER MARCET, M. D. F. R. S. one of the Physians to Guy's Hospital.

The patient had swallowed six ounces of laudanum; five hours after he was forced to swallow a drachm and ½ of sulphate of zinc, by which he was made to vomit about half an ounce of a fluid smelling strongly of opium. The symptoms increasing to an alarming degree, about half a drachm of sulphate of copper was dissolved in water. By great diligence of the attendants half this quantity was swallowed. This produced a copious vomiting, and afterwards, by continually moving and rousing the patient, he gradually recovered.

---

Reports

*Reports on the Effects of a peculiar Regimen on Schirrous Tumours and Cancerous Ulcers:* by WILLIAM LAMBE, M. D.

(Concluded from our last, pp. 508—516.)

" By parity of reasoning, [never was a happier expression,] continues Dr. Lamb, we ought to conclude, that the symptoms, which did not yield to this change of regimen, but which continued to recur with equal violence, though at irregular intervals, were wholly unconnected with the use of water; such are the sickness and vomiting, which have been always observed to form a striking feature in the constitutional symptoms of Cancer; the pains of the limbs; and the dropsical swellings."

These latter symptoms it is supposed must arise from something taken into the stomach, and this something, it is concluded, must be the solid matter, or rather that part of it which consists of animal food. From this time, therefore, a strict adherence to vegetable food is added to the distilled water regimen.

The second case was of an inmate in St. Andrew's workhouse, who at the age of 64 had had a cancer in her breast for twelve months. For three years she had suffered such pains in her limbs as rendered her a cripple. The progress of the breast had been from pricking pains to a lump, to which the skin adhered, which became discoloured and blistered on the surface. In the course of six or seven months other blisters arose, which running together formed a scab.

" July 13, 1805; at this time the scab is lower than the surrounding skin, and so much detached from it, that a probe might readily be passed between them. From this part it bleeds occasionally. Its situation is a little above the nipple. Still higher, and more towards the sternum, there is a lump, irregular and knobby on the surface, with an edge that is irregular likewise. The skin is discoloured over it, and of a dusky red. There is no tumour in the axilla, but there is pain in the arm, about the insertion of the deltoid muscle. The other breast is prettty full, and to appearance sound.

" Of the general health, there was no particular complaint, but a general debility; she complained of a sense of tightness across the stomach; the countenance was pallid, and the lips dark coloured, approaching to purple. Considering therefore her time of life, and general decrepitude, there was little hope of rendering her any essential service. The case could not be properly called open cancer; but there being a considerable breach of the substance of the skin, it was obviously fast approaching to that state. Wishing to see the genuine effect of pure water on such a case, I prepared it for her myself, and she used it regularly during the remainder of her life."

From this time to the middle of October 1807, the appearances are registered with an exactness which does equal credit to the author's patience, exactness, and candour. They remind us of what we we have too often seen, where the diet was unrestricted. In cases where the disease occurs at a very advanced age, and the progress in the course of a twelvemonth was no more than those met with by Dr. L. at his first interview with this patient, we should expect exactly such a progress and issue as he has related. Alternate vesications, serous and purulent discharges, ulcerations, fungating and sloughing processes, nor should we be at all surprized at the occasional skininng of the parts.*

The 3d case was of a woman 50 years old ; three or four years after the extirpation of her whole breast, a tumour about the magnitude of a filbert, appeared on the lower part of the sternum. It was of a livid red colour. After a time it sloughed out, leaving the surrounding skin with a smooth regular edge, as if cut with a knife.† She had cough ; difficult respiration ; seemed emaciated ; pulse about 120 ; but muscular strength not greatly impaired. In such a case as this, our readers, accustomed to view cancer, will hear without surprise, that she lived 13 months, that is, the commencement of the succeeding winter. But for her pulmonary symptoms we see no reason why she should not have lived longer on the customary diet of a workhouse, assisted by her friends, as this woman was. Her only restraint seems to have been in confining herself to distilled water.

The 4th was a rapid case, in a woman 36 years of age ; she died in a short time after the course was begun. The disease however did not spread under the regimen, but, on the contrary, the colour and appearance of the margin sensibly improved.

The

---

* The following account of the progress of a carcinoma is extracted from Dr. Adams's Correspondence with Dr. Baillie, and others, published in the year 1801. Page 106 alibi.

"When ulceration has begun, it is not as in common abscess, because matter has approached the surface with a previous elongation and as we call it pointing of the skin. For the fluid, which consists only of hydatids altered in their form by the loss of life, makes no progress towards the surface, but remains till the gradual sloughing and ulceration of the fungus expose the cavity in which they are contained. When by these means the fragments of the cysts and turbid fluid into which the hydatids are converted by death escape, the sides of the cavity do not collapse like that of common abscess, but expose a ghastly cavern, from which, instead of pus, a watery discharge is secreted, attended with a very peculiar smell. Round the mouth of this cavern, fungus sometimes grows, and instead of any attempt to unite, the skin curls in a contrary direction, which increases the depth of the cavity. This continues as long as any fragments of hydatid-tunics retain their life. As they die, ulceration takes place to detach them, like other extraneous bodies; and this ulceration being unattended with fungus, renders the cavity for a time still deeper. After all the fragments of tunics are detached, the wound will perhaps continue stationary or heal, or more commonly another set of hydatids will be so far advanced as to produce a fresh sloughing or ulceration."

† Compare this with Justamond's case treated with arsenic.

The 5th was also a rapid case. The patient lived from Oct. 1806, to the January following, under the regimen of distilled water; during this whole time the appearance of the cancer improved.

The sixth case was the effect of a blow. It remained about a twelvemonth, without any breach of the skin. Half a year afterwards Mr. Abernethy proposed the use of distilled water, and at the same time introduced Dr. Lamb to the case. It was then conjectured by Mr. Abernethy, that the patient could not live more than a twelvemonth at most. Of the symptoms at the latter end of May, 1805, we shall only remark, that the fungus was so considerable as to project greatly beyond the natural figure of the breast; the pain had never been acute. The general health, the author remarks, was as little affected as could be expected. She had however many symptoms of reduced health.

For the first eight months after commencing the distilled water, every thing promised well. On the ninth some unfavourable symptoms induced the author to propose the adherence to vegetable diet in addition to the distilled water. The progress of the disease varied from this time as well as the state of health; but the latter seems gradually to have declined. In the spring of the third year, small doses of tinct. ferri muriati were of great service. When they were begun, or how long continued, we are not informed. The patient lived till the October twelvemonth following.

The 7th case describes a widow 60 years old, who for *many years* had a tumour in her breast, which was originally caused by a pinch. Of the appearance of the disease we shall only remark, that the breast was smaller than the sound one, that the health was sound till she commenced the distilled water. In about five or six months a quantity of serous discharge took place, as had formerly been the case. At the end of a twelvemonth the tumour was either wholly or nearly absorbed.

The following are our author's remarks on this case.

"She left off the distilled water when the tumour was absorbed because she said that, she had never felt well, as long as she used it. She concluded, therefore, naturally enough, that it disagreed with her and did her harm. Upon resuming the use of common water, these uneasy feelings immediately disappeared. So great is the difference upon the body between this fluid in its common state, and when freed from all heterogenous matter; and so much are we the slaves of habit, which renders the most noxious irritations, to which we have been accustomed, necessary to our comfort.

"I find that still there is some uneasiness about the part which the tumour occupied, though it is slight. This circumstance proves, I think, that the nature of the disease has not been mistaken; and I have little doubt, that it will still prove troublesome, if she be not cut off by internal disease."

Many of our readers will be of opinion that the reduced health in consequence of distilled water, or from some other cause, might have produced absorption of tumours which never seem to have shown a destructive tendency.

The 8th case affords no satisfactory conclusion either for or against the regimen.

Case ix. we suspect would be called by the author we before alluded to, lymphatic hydatids, to distinguish them from the true carcinomatous. The former he considers as vesicles filled with fluid, the latter with fat. Dr. Lambe's subject was a widow, who for some years had perceived a small lump, which appeared just above the nipple.

"During the year 1805, the tumour increased, and in February 1806, a small hole had formed in the skin, which had become discoloured, and there was a fetid matter discharged from it. At this time the regimen I have so often spoken of, was recommended to her, but it was not adopted: and I know not what occurred from this time till the beginning of June 1807, except that the ulcer never closed, but continued to discharge a serous fetid matter; once the whole inflamed, and a number of oval vesicles came out; afterwards there was a discharge of a cream coloured matter. After this the ulcer contracted greatly; it, however, never closed, but enlarged by the gradual destruction of its margin."

This is what the same advocate for the hydatid nature of carcinoma would call the death and subsequent separation of a cyst of lymphatic hydatids. About a twelvemonth afterwards, the same train of circumstances took place as before the adoption of the regimen, viz. the parts inflamed, a quantity of oblong vesicles sloughed out, and afterwards a cream coloured fluid was discharged; "the ulcer then contracted." On a subsequent occasion, something like the same process was renewed, excepting that the vesicles came away gradually in pieces of about half an inch long, and as thick as a quill. In consequence of which the cavity was increased, and a cream coloured fluid discharged. The process was attended with much fœtor. The discharge gradually abated, the ulcer contracted and healed.

All these nine cases, as far as we can perceive, have followed the order described by the most accurate writers. If the process has begun early in life without a previous injury, the consequence has been so rapid a progress as has baffled every remedy, and outrun every operation.*

Dr. Adams, after reciting the success of different authors, concludes with the following remark.

"From these cases it seems to follow, that all the success practitioners have hitherto flattered themselves with, depends on the slowness of the progress of the disease. Thus we find that though Justamond's and Dr. Ewart's cases both healed for a time, yet Storck's and Mr. Simmons's, though mild, were too rapid to be ever

* Observations on Cancer, by Dr. Adams, above referred to.

ever completely skinned over after they were once ulcerated. Wiseman gives three cases of this kind, which he calls schirrhous cancers. The first healed for a time; the others continued for many years without much inconvenience to the patients. Thus it generally happens that the cases which promise the greatest success are old cancers, or cancers in old people, which though held out as the most formidable, are in reality the most innocuous.—Formidable they may be truly called, inasmuch as they place the disease beyond a doubt; but their former progress, or the late period of their commencement, evinces a comparatively less aptitude in the constitution; so that unless a change takes place in the general health or external circumstances of the patient, we have reason to hope that life will not be much shortened, and only occasionally embittered by this incurable malady."†

Such are the cancerous cases related by Dr. Lambe. A case of scrofula follows in a boy 11 years old. In this the author's success induces him to conclude, " that the scrofulous ulcer is prevented from cicatrizing, merely by the constant irritation of common water." Surely they do sometimes cicatrise under the use of common water. Many ingenious and powerful arguments follow, but whether they will prove more convincing to others than ourselves, it is not for us to determine.

" It is in vain, says our author, that I hear it objected, that chemistry can detect no difference between the blood of the herbivorous and the carnivorous animal. Be it so;—your chemistry then is imperfect. Some centuries more may elapse, before you arrive at a just analysis of vegetable and animal matter; and before you can ascertain the difference between blood, or bone, or muscle, formed from vegetable, and from animal food. It may be no more than a slight variation of the proportions. Alcohol and sugar are composed of the same elements. I shall not believe, on that account, that the former is wholesome nutriment, and the latter a deadly poison."

A case of long continued and obstinate asthma follows, related by the patient, which has been so effectually cured by distilled water and vegetable diet, that the writer has been induced to adopt a similar plan for his children, " than whom no children can possibly be healthier."

Such is the abstract of this valuable book; valuable we call it, because the whole is replete with internal evidence, which scarcely wants even the character of the author to confirm it. Every physical fact is valuable. But we conceive it will be unnecessary for us to give any further opinion of the success of the practice proposed.

---

† Id. p. 135.

Report

